# NIR Monitoring of Ammonia in Anaerobic Digesters Using a Diffuse Reflectance Probe

**DOI:** 10.3390/s120202340

**Published:** 2012-02-21

**Authors:** Chitra S. Raju, Mette Marie Løkke, Sutaryo Sutaryo, Alastair J. Ward, Henrik B. Møller

**Affiliations:** 1 Department of Engineering, Aarhus University, Blichers Allé 20, Tjele DK 8830, Denmark; E-Mails: sutaryo.sutaryo@agrsci.dk (S.S.); alastair.ward@agrsci.dk (A.J.W.); henrikb.moller@agrsci.dk (H.B.M.); 2 Department of Food Science, Aarhus University, Kirstinebjergvej 10, Årslev DK 5792, Denmark; E-Mail: mettem.loekke@agrsci.dk

**Keywords:** NIRS, biogas, ammonia, inhibition, monitoring, manure, PLS, iPLS

## Abstract

The feasibility of using a diffuse reflectance probe attached to a near infrared spectrometer to monitor the total ammonia nitrogen (TAN) content in an anaerobic digester run on cattle manure was investigated; as a previous study has indicated that this probe can be easily attached to an anaerobic digester. Multivariate modelling techniques such as partial least squares regression and interval partial least squares methods were used to build models. Various data pre-treatments were applied to improve the models. The TAN concentrations measured were in the range of 1.5 to 5.5 g/L. An R^2^ of 0.91 with an RMSEP of 0.32 was obtained implying that the probe could be used for monitoring and screening purposes.

## 1. Introduction

Intensive farming methods generate large amounts manure that need safe disposal. In Denmark, more than 33 million tonnes of manure are produced per annum [1]. Current manure management strategies involve spreading of manure on agricultural fields to recycle the nutrients, aerobic treatment, separation of the solid and liquid fractions, composting and anaerobic digestion among others [2]. Denmark has many full-scale biogas plants that use livestock manure as substrate along with organic wastes from industries [3]. Livestock wastes contain ammonia, which is inhibitory to anaerobic digestion, and contain compounds like urea and proteins that will degrade into ammonia [4]. Ammonia is present in the form of the ammonium ion (NH_4_^+^) and free ammonia (NH_3_), of which the free ammonia (FA) is suspected to be the main cause for inhibition [5]. The ammonium ion and free ammonia exist in equilibrium, and the equilibrium depends on the temperature and pH. A decrease in pH reduces the amount of free ammonia. When a process is inhibited by free ammonia, the methanogens are affected, and consequently the volatile fatty acids (VFA) accumulate reducing the pH, this in turn reduces the free ammonia concentration [4]. This leads to a stable condition but at a sub-optimal level called an inhibited steady state. Total ammonia nitrogen (TAN) levels of more than 4 g N/L were found to cause inhibition; levels beyond this showed stable biogas production after an initial adaptation period but this biogas yield was lower than that of uninhibited reactors [4]. Livestock manure can often have more than 4 g N/L of ammonia, especially in the case of swine manure and poultry manure [4], the ammonia concentrations can also be high in anaerobic co-digestion plants that mix high protein wastes to their substrates. Thus, monitoring the ammonia content of the slurry in anaerobic digesters is an important aspect of process control and in managing the substrate feeding rate.

Ammonia content is usually measured and monitored by laboratory analysis such as colorimetry. This procedure involves the use of reagents, is time consuming and is not practical for process control. Near infrared (NIR) spectroscopy has been used to monitor various process indicators in the anaerobic digestion process. Earlier experiments using Trans-flexive NIR Spectroscopy (TENIRS) has shown good results in predicting the ammonia contents in an anaerobic digester [6]. Another study that showed good results in applying NIR spectroscopy to predict ammonia, used the polyethylene bag method where a sample of cattle manure was filled into a polyethylene bag and then pressed on to the surface of the scanning window of the NIR spectrometer [7]. However, the TENIRS requires the use of a macerator to reduce the size of the slurry particles to below 3 mm before the sample can be sent through it. The polyethylene bag method requires the sample to be taken out of the reactor and then analysed elsewhere.

A previous study used a reflectance probe to monitor the propionate contents of a small continuously stirred reactor successfully [8]. It has also been shown that the probe can be directly fitted on to a reactor without major changes to the reactor body [9]. The aim of this study was to investigate the feasibility of predicting the ammonia content of manure using a diffuse reflectance probe. The reflectance probe also offers easy maintenance and unlike transmission spectroscopy does not depend on the transmission path lengths. A drawback of the reflectance probe is that more scatter is expected, and therefore more noise will be added to the spectra. This study describes the first step which is to determine if the diffuse reflectance probe can actually be used to determine the total ammonia nitrogen (TAN) concentrations in a complex material such as slurry from an anaerobic digester and if feasible, future studies can be directed at fitting the probe onto a full scale anaerobic reactor and test its performance. The slurry samples were scanned offline, using the diffuse reflectance probe. The spectral data was analysed using multivariate analysis and models relating the spectral data to the TAN contents were developed. Manure or slurry samples are a matrix of particles of different sizes and as a consequence, measurements based on reflectance mode will have variation in light scattering (*i.e.*, wavelength dependent path length variation) between samples which can negatively affect the modelling process. The effects of scatter can be corrected mathematically using data pre-processing methods; such methods were used to improve the models.

## 2. Experimental Section

### 2.1. Sample Collection

**Figure 1.**
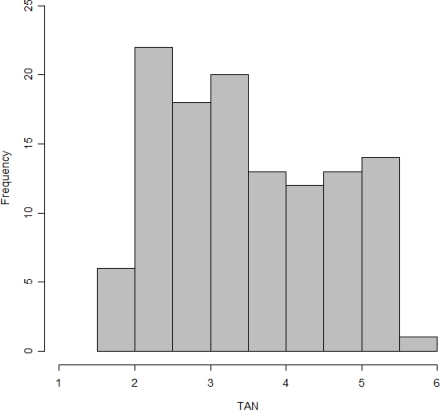
Histogram depicting the spread of the TAN values.

Five bench scale continuous reactors were run on cattle manure that was collected from dairy cattle farms located in the Research Centre–Foulum (Denmark). The reactors had a working volume of 7 L and were operated at a thermophilic temperature of 50 °C. Four of the reactors were used to test the effect of ammonia inhibition on the methane yield while the remaining one served as the control. Urea (crystallized Ph. Eur Cat. No. 2880.362) at concentrations of 0.175, 0.350, 0.525, 0.700% w/w were added to the four reactors to induce ammonia inhibition. The reactors had a retention time of 14 days. About 200 g of digestate was collected from each of the reactors twice a week, and the total ammonia nitrogen (TAN) content of the samples in g/L was measured by colorimetry at 690 nm, using the Spectroquant ammonium test 1.600683(EPA 350.1) and a Merck^®^ spectrophotometer After the TAN analysis the samples were frozen in 250 mL polyvinyl chloride (PVC) containers until the NIR scanning was performed. The TAN values were spread between 1.5 to 5.5 g/L and included many samples that had TAN levels more than the 4 g N/L above which process inhibition is said to occur [4]. These ranges were useful to see if the probe could detect ammonia at levels that are inhibitory and also at levels that are acceptable. Figure 1 is a histogram of the TAN values showing the spread of the reference data points. The effect of the ammonia inhibition on the methane yield of the manure will be published in a separate paper.

### 2.2. NIR Scanning

The NIR scanning was performed using a Bomem QFA Flex Fourier Transform spectrometer fitted with an InGaAs detector (Q-interline A/S, Copenhagen, Denmark). The diffuse reflectance probe that was used (QIA2050, also from Q-interline A/S) had a stainless steel body with a 5 mm sapphire window embedded into it and scanned in the range of 833.3 nm to 2,500 nm (12,000 cm^−1^ to 4,000 cm^−1^). The probe is specifically optimized for materials with high scatter like slurry from anaerobic digesters.

The NIR scanning, as mentioned earlier, was performed offline. The frozen digestate samples were first brought to room temperature (19 to 20 °C) by thawing at room temperature overnight. The NIR probe was rinsed with de-ionized water, wiped clean with a tissue and then placed into the PVC container containing the digestate sample and clamped into position using a laboratory clamp stand such that there was at least 2 cm of sample beneath it, ensuring that the position was the same for every sample. An agitator was immersed parallel alongside the probe and the digestate sample was mixed at 190 rotations per minute (rpm) to make sure that enough sample passed in front of the scanning window of the NIR probe. The speed of the agitator was optimized by trying different rpm settings to ensure that there was no bubble formation which would negatively impact the scan while ensuring the sample did not settle. For each sample, the measurement took about 80 s and consisted of 256 scans which were then averaged for that particular sample. A total of 119 manure samples were scanned in a time-span of two days. The background scan was measured against a white spectralon disk.

### 2.3. Model Calibration and Validation

NIR spectra are often noisy [10] due to various reasons, including instrument noise and high absorbing materials, and detector performance. The entire spectra, obtained from the NIR spectrometer amounted to 1,006 spectral variables. A lot of the variables were noisy due to high absorbance in wavelengths above 1,800 nm and due to low detector sensitivity to wavelengths below 900 nm. These areas were consequently cropped. There is often offset and slope variation between NIR spectra of samples that have equal analyte concentration but different light scattering properties. Light scattering differences in spectra can be minimized by data pre-processing.

Data pre-processing is therefore a necessary step before modelling and can be classified into two main types. The first are scatter correcting methods such as multiplicative scatter correction (MSC), extended MSC (EMSC), standard normal variate (SNV), de-trending, baseline offset correction (BOC) and normalization. The second are spectral derivative pre-processing methods such as Norris-Gap (NG) and Savitzky-Golay (SG) polynomial derivatives. The data pre-processing methods available in the Unscrambler Version 9.8 software were applied to the spectral data and using the pre-processed data, models to predict the TAN content were developed.

Two modelling methods: Partial least squares regression (PLS) and interval partial least squares (iPLS) were used to relate the spectral variables obtained from the NIR to the reference variable (the measured TAN values).

The commercially available Unscrambler Version 9.8 software (CAMO Software A/S, Oslo, Norway) was used to develop the PLS models. The PLS is based on the regression method developed by Herman Wold [11]. Each model was validated by both full cross validation and test set validation. Full or leave-one-out cross validation is a model validation method where one sample is left out iteratively and a calibration model is built, and then the sample that was left out is predicted using this model. The iteration is continued until all samples are left out of the calibration set once. For the test set validation the data set was divided into a calibration dataset and a validation dataset that both covered the range of ammonia levels: the data was listed according to the ammonia level, and every fourth sample was added to the test set (29 samples) and the rest of the samples were included into the calibration set (90 samples).

The other method used for modelling was the iPLS which is a graphically oriented local modelling procedure [12]. The iPLS builds local models on sub-intervals of the whole spectrum and selects the optimum sub-intervals in the spectral data to give precision prediction models [12,13]. Each sub-interval contains a selected number of spectral variables. The iPLS models were built using the PLS toolbox Version 6.2.1 (Eigenvector Research Inc., Wenatchee, WA, USA) in Matlab Version 7.12 (MathWorks, Natick, MA, USA). The iPLS was run using forward selection on raw and on SNV pre-processed spectra with a sub-interval size of 30 variables and a maximum of four PLS components (or latent variables) were allowed. To decide upon the number of components that could be used, the number of components was varied and looking at the RMSEP of the full model it was found that there was no advantage in using more than four components. For validation during iPLS optimization, full cross validation was used. When the optimum interval combination was found, the model was validated using the test set.

The prediction performance of the models was evaluated based on their modelling parameters: the coefficient of determination (R^2^), the root mean square error of prediction (RMSEP) and by their residual prediction deviation (RPD) which is the ratio of the standard deviation to the RMSEP [14]. The number of principal components used to construct the model was also used as an indicator. High R^2^ and RPD values, minimum number of components possible and low RMSEP values indicated a good model.

In general, eliminating redundant variables and basing models on the variables that are significant will give lower estimation errors [15]. The iPLS automatically provides the variables that correlate the most to the reference variable. In the case of PLS, once the best possible model was built, the number of spectral variables was reduced by using Marten’s uncertainty test function [16] to see if the model could be improved further by removing variables that are not important to the model. The uncertainty test function is available in the Unscrambler Version 9.8 software and uses the jack-knifing method to separate the unimportant variables from the useful ones hence simplifying the model. The reduced set of variables was used to build a new model and then the uncertainty test was once again used to reduce the variables further. This iterative approach was continued till the modelling parameters began to deteriorate. The uncertainty test was also applied on the entire spectral range to investigate if the results, after removing the unimportant variables, would be comparable to those of the best models obtained by other methods.

## 3. Results and Discussion

The validation statistics including the modelling parameters of selected models are listed in Table 1. Models 1 to 6 are the PLS models while models 7 and 8 are from iPLS. From the Figure 1 it can be seen that the frequency of the TAN values is not even. An even spread of values would give a more robust calibration model [17].

**Figure 2.**
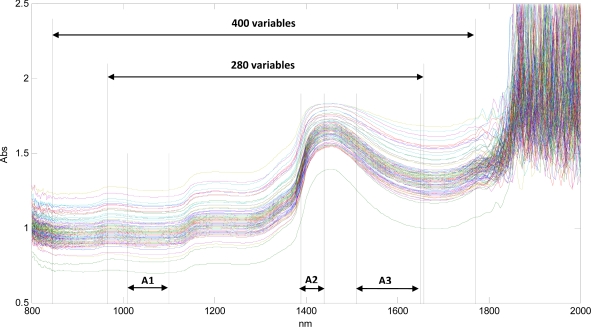
Spectral regions used for developing the models. A1, A2 and A3 represent the regions associated with the NH_4_^+^ group.

Figure 2 is a spectral plot of the absorbance *vs.* the wavelengths (in nanometers) for all scanned samples and gives an overview of the various spectral regions used for constructing models in this study. The raw data obtained from the NIRS included noise, and the noisy sections of the spectra were removed after visual inspection and reduced to 400 variables in the region of 847.2 nm to 1,770.8 nm. At the same time, the plot of the raw spectra showed one scan that seemed very different from the others, and this scan was removed as an outlier. Three other samples with high residuals and Hotelling T^2^ values were also excluded. An example of the results plot obtained from the PLS modelling done using the Unscrambler Version 9.8 software, is shown in the supplementary section.

**Figure 3.**
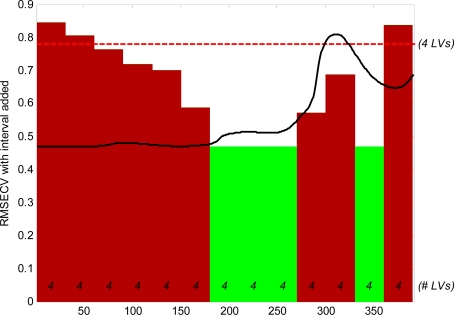
The output of iPLS; RMSECV with intervals *vs.* the selected variables (represented as variable numbers, not wavelengths). The selected variables are in green and the omitted variables are in red. The number of latent variables (LV) used are shown as well.

Model 1 is based on the 400 variables, the relatively low R^2^ and high RMSEP showed that there was scope for improvement. Various continuous sections within this selected spectral region (with 400 variables) were investigated and the region that gave the best correlation (model 2) was selected for further data pre-processing. The spectral region that gave the best correlation to TAN was between 967.3 nm to 1,657.6 nm and included 280 variables. This region includes most of the regions associated with the NH_4_^+^ group. The wavelengths in NIR spectroscopy associated with the NH_4_^+^ group are: 1,010 to 1,100 nm, 1,390 to 1,440 nm, 1,510 to 1,650 nm (represented as regions A1, A2 and A3 respectively in Figure 3) and 2,330 to 2,400 nm [18]. However, model 5 which was built by selecting only the specific spectral regions associated with the NH_4_^+^ group (excluding the last range which was noisy) and correlating it to the reference variable did not perform better than model 2 or model 3. In NIRS, anharmonicity, interactions between the constituents [18] and overlapping absorption bands make it difficult to ascribe a particular component to a certain wavelength region. The use of chemometrics and especially multivariate variable selection methods such as jack-knifing and iPLS make it possible to overcome this by identifying the variables that are most relevant. A model based on all the 1006 spectral variables available, showed extremely low R^2^ and extremely high RMSEP which was expected as a lot of noisy variables had been used. But reducing the number of variables to 43 by using the uncertainty test iteratively, improved the prediction capabilities of the model based on the entire spectral range considerably (model 6) again emphasizing the importance of choosing the right variables.

While using PLS modelling on the 400 variables dataset, pre-processing of the data improved the R^2^ and RMSEP of the models only slightly. Pre-processing the spectral data (280 variables) by the SNV method (model 3) improved the model slightly more than other pre-processing methods. SNV is used to remove slope variation and to correct for scatter effects. This is a mathematical transformation method, where each spectrum is corrected individually by first centering the spectral values, and then the centered spectrum is scaled by the standard deviation calculated from the individual spectral values [10,19]. Although the improvement in the model was small, the number of components used for the modelling decreased by 2. Reduction of the number of components used in modelling increases the robustness of the model and makes the model less sensitive to noise [15]. Lowering of the number of components indicates reduction of noisy variables that are included in the calibration model. This was similar with the use of the uncertainty test to reduce the number of variables (model 4). Although model 4 did not change much in terms of R^2^ and RMSEP compared to model 3, the number of components was reduced by 3 and the number of variables used to build the model were reduced considerably.

Figure 3 is a sample of the graphical output from the iPLS modelling, which is a plot of the RMSECV *vs.* the variable number (model 8). It visually presents the variables that were most relevant for the modelling process by selecting the intervals that have a low RMSECV and indicating them in green and the redundant ones in red. The dotted line at the top of the plot represents the RMSECV of a model built on the entire variable range. A plot of the mean spectra is also given as a black line which aids in identifying the regions of the spectra that are important.

The iPLS procedure was used as another method for selecting the optimum variables regardless of knowledge of the assignment of the NH_4_^+^ group in the NIR region. The iPLS models used the 400 variables region to iteratively search for variables that gave the least RMSECV. The iPLS models improved in terms of R^2^, RMSEP, and the number of components when data pre-processed by SNV (model 8) was used compared to the iPLS model using data that was not pre-processed (model 7). Interestingly the noise reduction due to the pre-processing step had a more pronounced effect on the model statistics in iPLS than in the PLS models that were based on the larger spectral range.

The iPLS model was based on a combination of the spectral intervals 1,127.2 to 1,333.6 nm, and 1,525 to 1,634.7 nm. The first range of optimal selected spectra does not correspond to the wavelengths normally associated with ammonia, but the selected spectral variable range 1,525 to 1,634.7 nm lies within the range of 1,510 to 1,650 nm which is associated with ammonia [18]. Comparing the modelling parameters obtained from PLS models and the iPLS models it can be seen that except for the number of components which are much lower in the iPLS model, the R^2^ and the RMSEP are quite close to each other.

Thus, based on all the models seen in Table 1, it is indicative that the spectra provided by the diffuse reflectance probe can be correlated to the TAN content. The iPLS model based on the data pre-processed by SNV gave an R^2^ of 0.91 and an RPD of 3.39, which is considered a successful model [20,21]. Future research is needed to test the probe in-line in the reactor and with an independent test set. Comparing this with other reported results; the TENIRS system uses trans-flexion, a combination of transmission and reflectance and requires the use of a transmission vial for the scanning process [22]. Transmission is usually used for spectral analysis of liquids while solids are scanned by reflectance [22]. Since manures and slurries are a combination of both liquids and solids, the inclusion of both transmission and reflectance could be an important factor for the R^2^ of 0.98 in the TENIRS experiment. One disadvantage of using a transmission vial for the measurements is that it is susceptible to clogging and to the formation of deposits. These lead to inaccuracies, due to a change in the transmission path length which is vital to the calculations involving the received signal. The diffuse reflectance probe does not have this problem.

High VFA concentrations cause inhibition, as the methanogens are sensitive to pH changes. Changes in VFA concentration is also indicative of process imbalances, as any inhibition of methanogens will lead to VFA accumulation. Previous studies using the diffuse reflectance probe in an anaerobic digester have shown that it can also be used to predict the VFA concentrations [8,9] and can thus be used to monitor VFA along with TAN. Apart from a monitoring system that could indicate inhibitory levels of TAN content, the NIR probe could also be used to screen manure based substrates for their TAN contents prior to loading into the reactor thus preventing the risk of inhibition. It can be used to maintain an optimum C/N ratio, between 20/1 and 30/1, which is another way of preventing ammonia accumulation and improving digester performance [23]. The use of NIR in predicting the amount of TAN will also aid in feed input management especially while dealing with feedstock that is high in protein and TAN content. In a previous study, NIR spectroscopy has been used to predict the biochemical methane potential (BMP) of meadow grass based substrates [24]. If further studies indicate that the diffuse reflectance probe can be calibrated to predict the BMP of manure based substrates, the probe could serve multiple purposes in the process control of anaerobic digesters.

## 4. Conclusions

The models obtained were successful and thus the diffuse reflectance probe is promising as an online ammonia monitoring tool for materials such as manure and digestates from anaerobic digesters. Based on the spectra obtained from the probe, PLS and iPLS gave similar models, except iPLS used lesser number of components indicating a more robust model. Pre-processing of the data also reduced the number of components in the models when compared to models that were based on raw data. Selecting the correct range of spectra that would be used in the model, however, proved to be very important in this process.

## Supplementary Information



## Figures and Tables

**Table 1.** Validation statistics.

**Model number**	**Method**	**Data pre-processing**	**Number of spectral variables**	**Spectral range (nm)**	**RMSECV**	R^2^ (CV *)	**Number of PCs**	**RMSEP**	R^2^ (TS **)	**RPD**
1	PLS	raw	400	847.2 to 1,770.8	0.66	0.63	6	0.56	0.72	1.93
2	PLS	raw	280	967.3 to 1,657.6	0.36	0.89	11	0.34	0.90	3.17
3	PLS	SNV	280	967.3 to 1,657.6	0.38	0.88	9	0.32	0.91	3.43
4	PLS	SNV	73	-	0.36	0.89	6	0.37	0.88	2.91
5	PLS	SNV	117	1,010 to 1,100, 1,390 to 1,440 and 1,510 to 1,650	0.45	0.83	16	0.50	0.77	2.17
6	PLS	raw	43	-	0.54	0.76	6	0.46	0.81	2.34
7	iPLS	raw	119	1,127.2–1,333.6 and 1,525.0–1,634.7	0.55	0.74	7	0.46	0.81	2.33
8	iPLS	SNV	119	1,127.2–1,333.6 and 1,525.0–1,634.7	0.43	0.84	5	0.32	0.91	3.39

* Cross validation;

** Test set validation; Model number 4 is obtained by reducing the number of variables used in model 3; The spectral ranges for models 4 and 6 are not mentioned as they consist of many discontinuous intervals.
