# Supplementary material for: NIR Monitoring of Ammonia in Anaerobic Digesters Using a Diffuse Reflectance Probe
Source: Sensors (Basel). 2012 Feb 21;12(2):2340–50. doi: 10.3390/s120202340 (PMC3304169; doi:10.3390/s120202340)
Supplement: Supplementary file 1 [file sensors-12-02340-s001.pdf]

Supplementary Information

NIR Monitoring of Ammonia in Anaerobic Digesters Using a Diffuse Reflectance Probe

Chitra S. Raju [1,*], Mette Marie Løkke [2], Sutaryo Sutaryo [1], Alastair J. Ward [1] and Henrik B. Møller [1]

[1] Department of Engineering, Aarhus University, Blichers Allé 20, Tjele DK 8830, Denmark; E-Mails: sutaryo.sutaryo@agrsci.dk (S.S.); alastair.ward@agrsci.dk (A.J.W.); henrikb.moller@agrsci.dk (H.B.M.)

[2] Department of Food Science, Aarhus University, Kirstinebjergvej 10, Årslev DK 5792, Denmark; E-Mail: mettem.loekke@agrsci.dk

* Author to whom correspondence should be addressed; E-Mail: chitras.raju@agrsci.dk; Tel.: +45-8715-7874.

Received: 18 January 2012; in revised form: 16 February 2012 / Accepted: 16 February 2012 / Published: 21 February 2012

Actual *vs.* predicted plot for model 3

Actual *vs.* predicted plot after full cross validation:

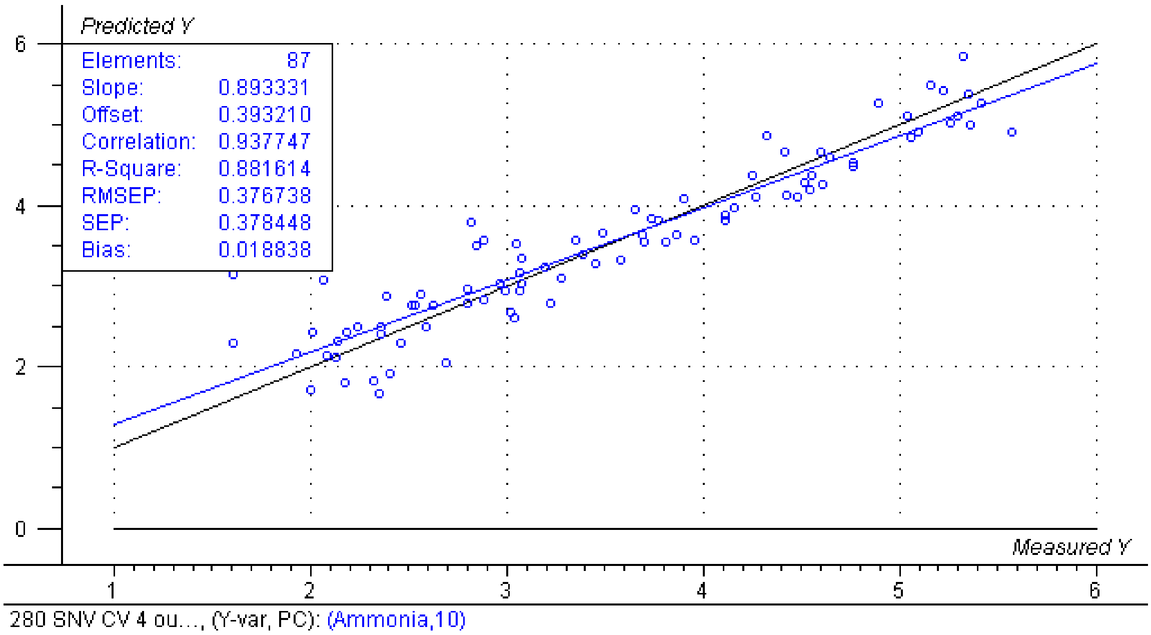

Actual *vs.* predicted plot after test set validation:

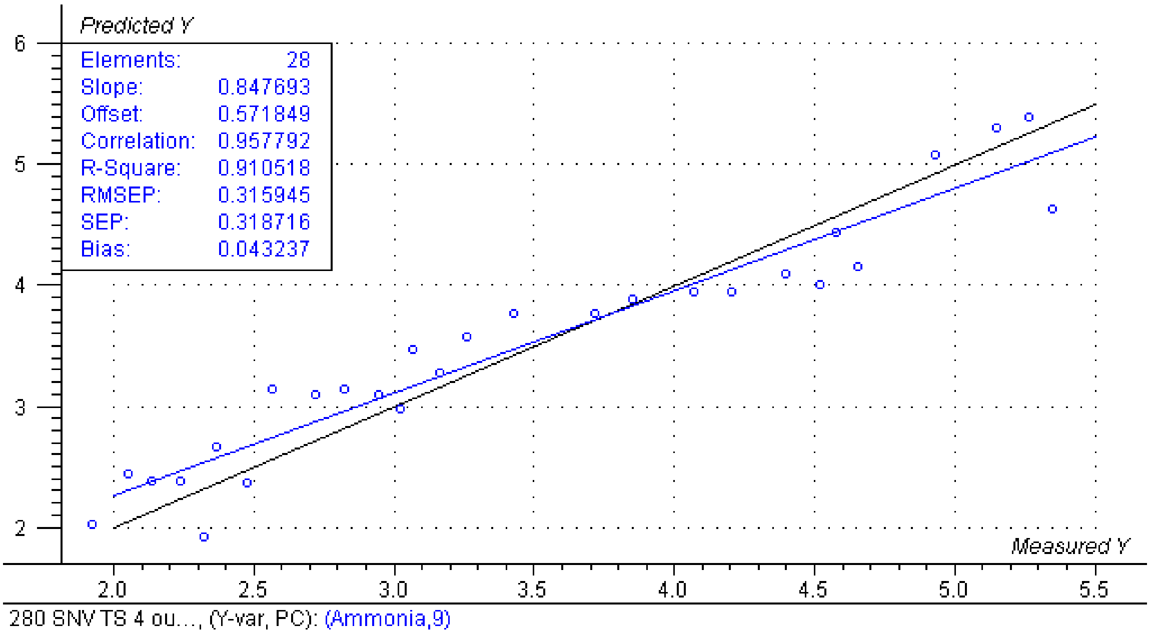

© 2012 by the authors; licensee MDPI, Basel, Switzerland. This article is an open access article distributed under the terms and conditions of the Creative Commons Attribution license (http://creativecommons.org/licenses/by/3.0/).
